# The Patient’s Record

**Published:** 1891-01

**Authors:** 


THE PATIENT'S RECORD. For the use of Physicians and Nurses. Compiled by AGNES S. BRENNAN. Quarto, pp. 100. G. P. Putnam's Sons, New York and London. The Knickerbocker Press. 1890.

This record has rulings for date, time, temperature, pulse, respiration, medicine, nourishment, stimulants, and remarks. It also has interleaved slips for entering the "doctor's orders." At the end of the book, bound in, but with perforated pages, are a number of temperature charts, with rulings for pulse, respiration, and urine in twenty-four hours, that serve a convenient purpose. It furnishes a neat way of preserving daily clinical histories, and will be found very useful in hospitals and clinics.

